# Orbital Extension of a Giant Ethmoidal Sinus Osteoma in a 30-Year-old Female

**Published:** 2013

**Authors:** Esmat Karbassi, Aliasghar Arabi Mianroodi, Ayeh Shamsadini

**Affiliations:** 1 *Department of Ophthalmology, Shafa Hospital, Kerman, Iran.*; 2 *Department of Otorhinolaryngology Head and Neck Surgery, Shafa Hospital, Kerman, Iran.*

**Keywords:** Ethmoid, Giant osteoma

## Abstract

**Introduction:**

Osteoma is a benign tumor rarely found in the paranasal sinuses.

**Case Report:**

A 30-year-old female presented with an 8-month history of proptosis of the right eye that was progressing slowly. She was diagnosed with ethmoid osteoma and managed with collaborative surgery at the ophthalmology and otolaryngology departments. After surgery, the patient suffered visual loss that was managed medically. The surgical steps and protocol used for safe removal are discussed in the report.

**Conclusion:**

Management of a giaint osteoma extending to orbital tissue needs meticulous dissection through open approach and collaborative surgery by otolaryngologist and ophthalmologist.

## Introduction

Osteomas are benign tumors rarely found in the paranasal sinuses (1) and are most frequently encountered in the frontal sinus, and to a lesser extent in the ethmoid and maxillary sinuses, and rarely in the sphenoid sinus (2). Frontal sinus osteomas account for 57% of all paranasal osteomas (3), with an incidence of 0.1 to 3% (4). Osteomas of the mandible, temporal bone, and mastoid have been described. The condition most commonly presents during the second to fifth decades, with a male to female ratio of approximately 3:1 (5). 

Paranasal sinus osteomas have a tendency to grow slowly, and for this reason they are generally asymptomatic and are diagnosed only as a coincidental radiological finding (3). An osteoma located away from the sinus ostium usually does not cause symptoms for a long time, although symptoms arise when the osteoma enlarges or is located in the drainage pathway of the sinus. Symptoms of ethmoid osteoma occur earlier than osteoma of the frontal sinus because of the small volume of the sinonasal cavity (6). Headache localized over the area of osteoma, facial pain or deformity, rhinorrhea, anosmia, and epistaxis are common symptoms (7). Extension to the orbital and/or skull base is unusual. When osteomas expand into the orbital vault, they displace the orbital contents and give rise to noticeable symptoms including headache as well as ocular symptoms such as diplopia, exophthalmos and proptosis (8). Histopathologically osteoma is hard and lobulated with an ivory-like appearance often mixed with a course granular component. The bone is compact or cancellous, with vascular or connective tissue components (7). 

Surgery is the treatment of choice for symptomatic ethmoid osteoma. However, the approach is under discussion and depends on the extension and occurrence of complications (8). Traditional surgical approaches include external rontoethmo- idectomy, lateral rhinotomy or osteoplastic flap techniques (9). In this paper we report a case of a huge ethmoid sinus osteoma with orbital expansion, treated with the Lynch approach.

## Case Report

*Case Description *A 30-year-old female was referred to our hospital with an 8-month history of proptosis of the right eye that was progressing slowly. She complained of double vision and pain around the right eye. Her previous medical history was unremarkable. During the previous 2 months she had experienced epiphora, positional headache, and painful eye movements, as well as nasal and post-nasal discharge. 

The ear, nose, and throat examination revealed only nasal and post-nasal discharge. Ophthalmologic examin- ations showed severe proptosis of the right eye anterolaterally (approximately 3 mm), mild diplopia, and painful motility. Her visual function was mildly disturbed and revealed diplopia on left gaze. Visual acuity was measured as 20/20 in each eye and intraocular pressure (IOP) was normal. Her eye movement had a slight adduction limitation – (−1 grade). 

Other movements were unaffected. The right globe was pushed downward and laterally. There was mild swelling of the right lower lid. 

**Fig 1:**
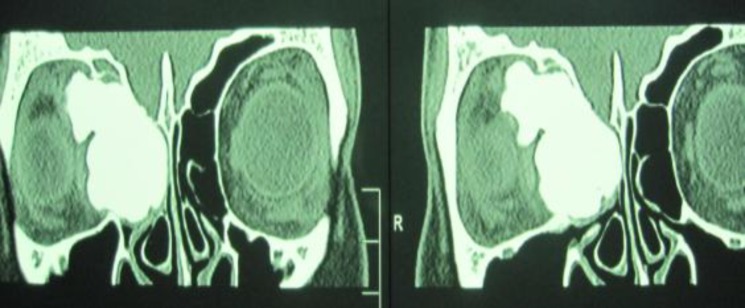
Coronal CT scan showing extension to the orbit

A hard nodular mass could be felt on and below the right medial cantus. Preoperative radiographic diagnostic work-up was axial, while a coronal computed tomography (CT) scan of the paranasal sinuses revealed a radiopaque mass in the right ethmoidal air cells with protrusion to the right orbit arising from anterior ethmoid sinus with extension upwards to the roof of the sinus and posterior ethmoid sinus and laterally in to the right orbital (Fig 1). 

The ophthalmic side of the tumor was not smooth, with an irregular surface and was posterior laterally close to the optic nerve. 

***Surgical technique***

We approached the tumor with a right-side Lynch incision. The endoscopic approach was not suitable as the lateral side of the tumor was deeply extended into the orbit. In order to gain better exposure, we removed part of the ascending process of the maxilla and the lacrimal bone. 

Because of the large size of the tumor, we divided it in two halves by drilling at the narrowest segment in an orbital and ethmoidal portion. 

**Fig 2:**
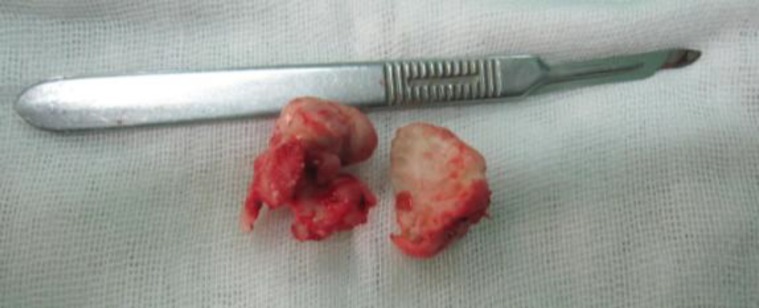
Specimen after excision in the two main sections of the tumor

The ethmoidal portion was excavated by drilling and then the remaining eggshell of the tumor was separated from the ethmoid roof to prevent damage to the skull base and possible cerebrospinal fluid (CSF) rhinorrhea. The orbital portion had an irregular surface (Fig. 2) and the superior oblique and medial rectus muscles were trapped in the tumor.

We had to divide the orbital portion into pieces by drilling and freeing the entrapped muscles before removing the tumor. We repeatedly monitored the optic nerve function by observing the pupil size. 

Post-operatively, the patient’s proptosis disappeared completely. She suffered some limitation of inward eye movement, as well as decreased vision to 5/10 due to macular edema. We initiated oral prednisolone therapy (1 mg/kg daily) for one week. The patient’s condition improved to normal over the course of 4 weeks. The patient was observed at follow-up and was symptom-free after 1 year. 

## Discussion

Although small frontoethmoidal osteomas are relatively frequent, giant osteomas are a particularly rare finding (10). Ethmoidal sinus osteoma tends to cause symptoms earlier than those in the frontal sinus because of the restricted space in the ethmoidal region and the consequent earlier encroachment on the neighboring structures. Extension to the orbit may lead to diplopia and proptosis (11). Lesions larger than 3 cm in diameter are considered giant tumors (12). Most authors, however, describe osteomas of a standard size. In a retrospective evaluation of 34 patients with frontoethmoidal osteomas, Schick et al reported a diameter ranging from 8 to 35 mm and a mean of 17 mm (5). Our patient showed typical ocular findings; i.e., proptosis and extra ocular muscle displacement (causing diplopia). 

Any surgical approach has to take into account protection of the vital structures, particularly the optic nerve and cribriform plate, complete resection, and minimal cosmetic deformity. Different approaches have been discussed in the literature for the management of large ethmoidal osteomas; including external frontoethmoidectomy, lateral rhinotomy, midfacial degloving, and osteoplastic flap. An endoscopic approach is used in selected cases (13). The most appropriate approach for a fronto-ethmoidal osteoma is selected according to location, tumor volume, side of the osteoma, anatomical situation including anteroposterior dimension, frontal recess, and extra sinus extension (13). 

The endoscopic approach has been used for this purpose recently. A number of reports in the literature describe the use of an endoscope to remove osteomas of ethmoidal origin, but none of these cases had an ophthalmic complication, while in all cases there was access to the tumor for endoscopic dissection (5,6).

Our case is made distinct by a number of ophthalmic complications due to extension of the tumor to the midsagittal plane of the orbit with a lobulated surface that was extended to the optic nerve and was entrapped into the surrounding fascia. Despite gentle manipulation on the posterior part of the tumor, mild ischemic neuritis of the ophthalmic nerve occurred that responded well to oral prednisolone (1 mg/kg daily) for 3 weeks. Traction of the optic nerve was an inevitable and regrettable consequence in our case as the orbital content was so trapped in the furrows of the tumor surface that we could not free the tumor until we removed it and saw that the orbital structures were on traction. 

The open approach is the most appropriate way to remove an osteoma extending into the orbit and the cooperation of both otolaryngologists and ophthalmologists is necessary to achieve risk-free surgery. 

## Conclusion

Treatment of large ethmoid osteomas with orbital complications requires meticulous dissection of the surrounding tissues through an open approach to prevent injury to the orbital content. Involving an ophthalm-ologist familiar with orbital surgery supports incident-free surgery.
